# Plain Talk about Insanity

**Published:** 1872-05

**Authors:** 


*Plain Talk about Insanity:* its causes, forms, symptoms, and the treatment of mental diseases. With remarks on Hospitals and Asylums, and the Medico-Legal aspect of Insanity. By T. W. FISHER, M. D. Boston: ALEXANDER MOORE, 1872.

This book comprises some very interesting "Plain Talk" in regard to Insanity which will be as valuable to the professional, as to the non-professional reader for whom it is principally intended.

The task of writing a book on any subject, strictly professional in its character which will be adapted to the popular mind, is one of considerable difficulty. In none of the many recent works of this character have we seen this task better accomplished than it has been by Dr. Fisher.

The causes, forms, symptoms, and treatment of insanity are each considered under its appropriate head, the Medico-Legal aspect of Insanity is treated of to some extent and a number of illustrative cases cited. Much of the matter for this portion of the work is derived from Dr. Ray's work.

Although some of the terms used would perhaps be unintelligible to non-professional readers, yet the work as a whole is of much interest and deserves a large circulation.

